# Open Hip Abductor Repair: Double-Row, Rip-Stop Technique

**DOI:** 10.1016/j.eats.2025.103802

**Published:** 2025-08-12

**Authors:** Jared J. Reid, Cody C. Ashy, Henry B.G. Baird, Erik J. Hansen, W. Michael Pullen

**Affiliations:** Department of Orthopedics and Physical Medicine, Medical University of South Carolina, Charleston, South Carolina, U.S.A.

## Abstract

Tendinopathy of the gluteus and minimus tendons is an increasingly recognized source of lateral hip pain. In recalcitrant cases, following exhaustion of nonoperative modalities, operative treatment is being considered more aggressively and optimistically with both open and endoscopic techniques, promising significant pain relief and gait improvement. Recent cadaveric biomechanical analysis demonstrated increased anatomic footprint coverage and higher load to failure of double-row compared to single-row suture anchor techniques. Because of the combined advantages of both double-row anchors and rip-stop repair demonstrated in rotator cuff repair, this article proposes and highlights an open, double-row, rip-stop suture anchor technique to address abductor tendon tears.

Gluteus medius and/or minimus (GMM) tendinopathy and tearing at the greater trochanter are increasingly recognized as causes of recalcitrant lateral hip pain (LHP).^1^, ^2^, ^3^ Women are disproportionately affected, experiencing pain and debilitation comparable to end-stage hip osteoarthritis.^4^ Histologic analysis of the trochanteric bursa in LHP patients often lacks acute inflammation but consistently correlates with abductor tendinopathy.^5^, ^6^, ^7^

Idiopathic GMM tears likely mirror the degenerative changes seen in rotator cuff pathology, leading to hip abduction weakness and Trendelenburg gait.^8^, ^9^, ^10^ Iatrogenic tears may result from prior hip or femur surgeries, including arthroplasty, femoral nailing, and iliotibial band procedures.^6^ Most cases initially undergo nonoperative management, including activity modification, anti-inflammatory treatment when feasible, and physical therapy to strengthen the abductors.^11^^,^^12^ Peritrochanteric cortisone or platelet-rich plasma injections, often performed under ultrasound guidance, may also provide symptom relief.^13^, ^14^, ^15^, ^16^, ^17^ Surgery is considered when conservative treatment fails, with both endoscopic and open repair techniques demonstrating pain relief and gait improvement.^3^^,^^18^, ^19^, ^20^

Endoscopic repair offers superior visualization of partial-thickness tears but is technically demanding, while open repair is preferred for full-thickness tears due to improved soft-tissue mobilization, visualization, and potential graft augmentation.^18^^,^^21^ Biomechanical studies suggest that double-row (DR) suture repairs provide superior footprint coverage and higher load to failure than single-row (SR) techniques, although no clear clinical consensus exists, as seen in rotator cuff repair.^22^^,^^23^ This technical note describes a DR, rip-stop technique for full-thickness GMM tears.

## Surgical Technique

After obtaining informed consent, the patient is brought to the operating room and given standard preoperative antibiotics. We prefer the lateral decubitus position to facilitate soft tissue retraction (Table 1, Video 1). Surgery can be performed under spinal or general anesthesia. Following induction, the patient is positioned laterally using a peg board or beanbag, and the operative side is prepped and draped, and a surgical pause is performed per hospital protocol. A padded Mayo stand is also available to support the foot in abduction to relieve iliotibial band (ITB) tension (Table 1, Video 1).

**Table 1:** Pearls and Pitfalls of Open, Double-Row, Rip-Stop Technique

Pearls	Pitfalls
Proper positioning / padding to relax ITB during surgical approach	Applying this technique without adequate mobilization of abductor tendons may over tension tissue, leading to failure of repair.
Mobilization of retracted tendon with Allis clamps or traction stitches with blunt-finger digital dissection around the tendons	Failure to relax the IT band with appropriate abduction may reduce visualization.
Footprint preparation with combination of Cobb elevator, curette, rongeur, and high-speed burr	Narrowed anchor spacing may induce fracture of the trochanter with poor bone mineral density.
Spacing of medial and lateral row anchors to promote maximum footprint coverage.	Suture management is critical to ensure appropriate rip-stop function.
Pass rip-stop suture first to ensure sequential suture tapes are passed proximally, increasing suture pullout strength.	
PEEK suture anchors are utilized more commonly rather than biocomposite to ensure maximum purchase in more dense bone of the trochanter.	

ITB, iliotibial band.

**Fig. 1:**
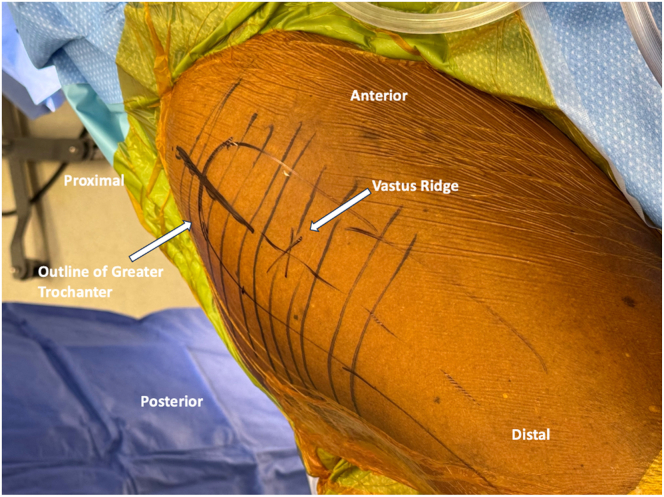
Superior view of the right, lateral hip with patient in the lateral decubitus position. The skin incision is centered just anterior to the prominence of the greater trochanter, measuring ∼10 cm in length. Lateral decubitus positioning facilitates soft tissue retraction during the dissection.

**Fig. 2:**
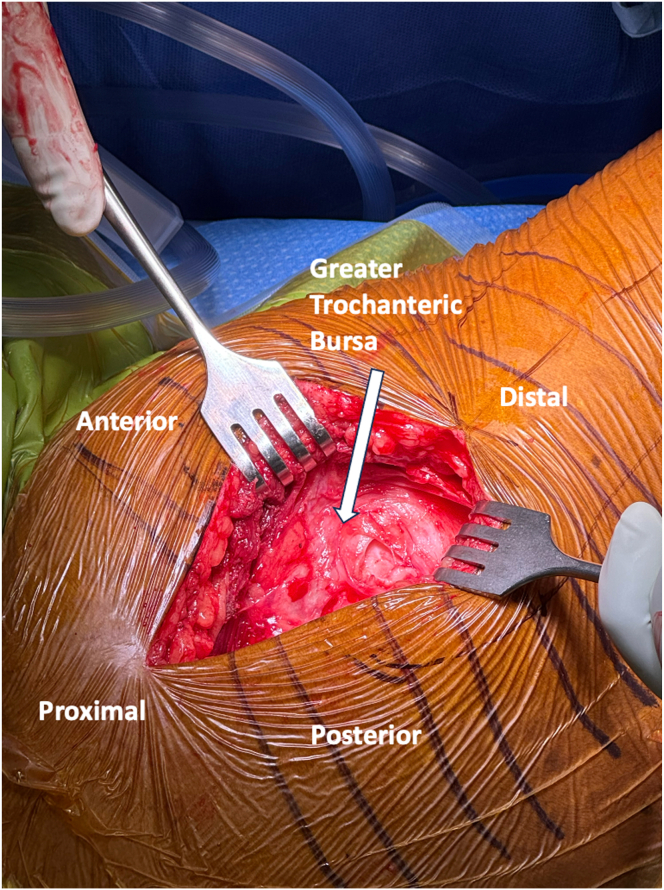
Superior view of the right, lateral hip with patient in lateral decubitus position. Iliotibial band incised longitudinally, in line with its fibers and logn axis of the femur, revealing underlying greater trochanteric bursa.

**Fig. 3:**
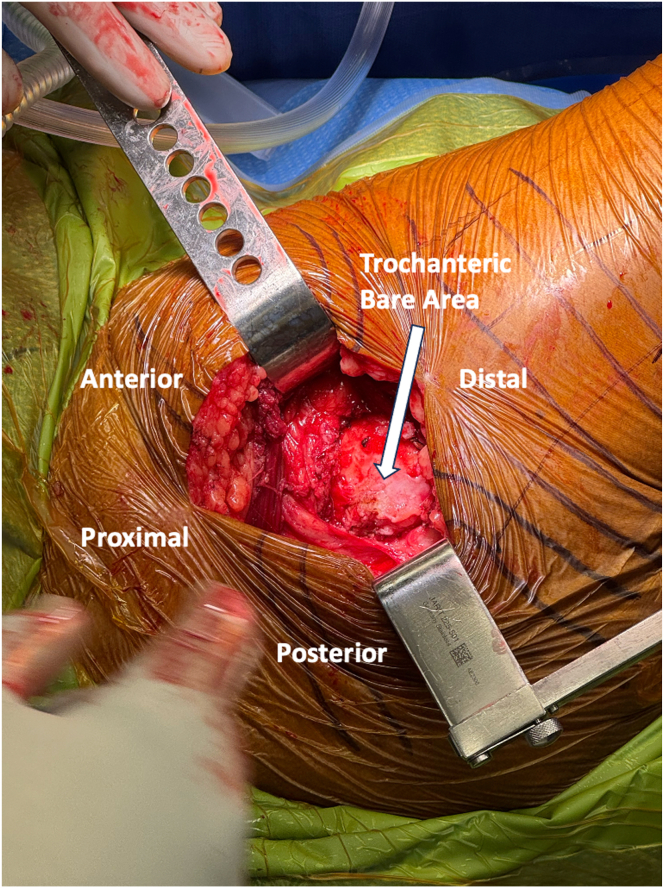
Superior view of the right, lateral hip with patient in lateral decubitus position, deep self-retaining retractor retracting iliotibial band anteriorly and posteriorly. Greater trochanteric bursa is excised, and deep, self-retaining retractor is subsequently placed, retracting iliotibial band anteriorly and posteriorly. Bare area of the trochanter is revealed, also with anterior, lateral, and superolateral facets of the greater trochanter in the setting of torn, retracted gluteus medius, and minimus tendons. Internally rotating the extremity in this position can facilitate visualization of the anterolateral and posterosuperior footprints of the gluteus medius and minimus.

A 10 cm longitudinal incision, centered over the greater trochanter (GT) extending distally 5 cm and proximally 5 cm, is made with a no. 10 blade (Fig 1, Video 1). Sharp dissection is continued through skin and subcutaneous tissue to the deep fascia and ITB. The ITB is incised longitudinally, in line with its fibers and the long axis of the femur, exposing the underlying trochanteric bursa (Fig 2, Video 1). Self-retaining, deep retractors are placed, and the extremity is internally rotated to visualize the anterolateral and posterosuperior footprints of the gluteus medius and minimus (Video 1). The GT bursa is excised, revealing the “bald” trochanter that is commonly encountered with large, retracted tears (Fig 3, Video 1). Mucoid, degenerated tissue is debrided to allow mobilization of the torn abductor tendons. In some cases, tearing may be underrecognized with gross inspection alone, yet ballottement of the tendons often reveals undersurface delamination that can then be incised and elevated off of bone. Saline injection beneath the gluteal tendon insertion may elevate the tendon undersurface rupture, commonly referred to as the “bubble sign.”^2^ An Allis clamp is used to mobilize the torn tendon edges to confirm adequate excursion for reparability and appropriate resting tension (Table 1, Video 1). Traction stitches are placed to aid in mobilization. The tendon footprints on the greater trochanter are then prepared with a combination of Cobb elevators, curettes, and rongeur to provide a bleeding bed of bone to facilitate tendon-to-bone healing (Table 1).

**Fig. 4:**
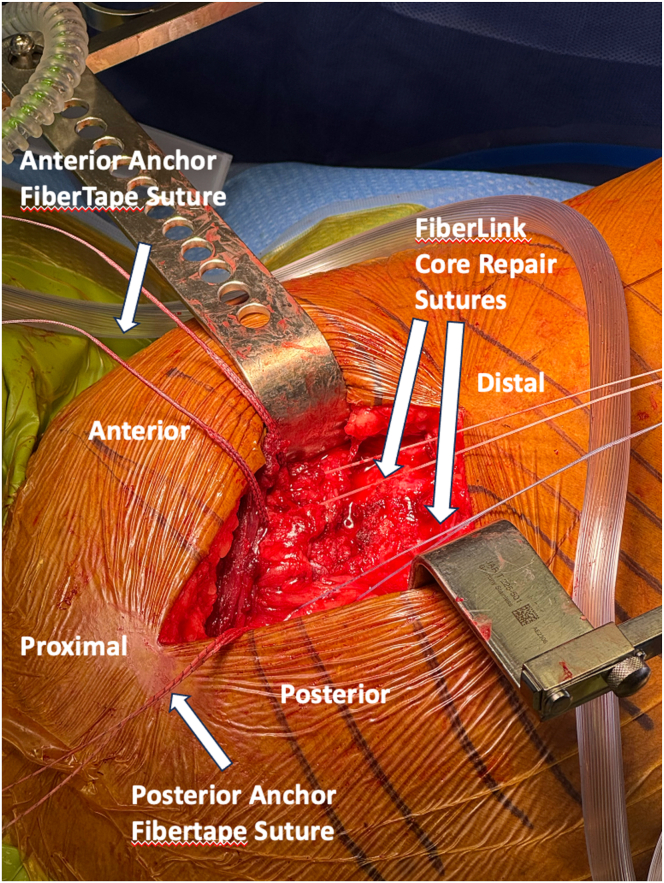
Superior view of the right, lateral hip with patient in the lateral decubitus position deep self-retaining retractor retracting iliotibial band anteriorly and posteriorly. Proximal row anchors, preloaded with FiberTape loops and FiberLink core repair suture (2 × 4.75-mm vented PEEK SwiveLock anchors), are placed. With a free needle, the FiberWire Core sutures are passed separately into respective gluteus medius and gluteus minimus tendons from superficial to deep, providing a horizontal mattress configuration to later serve as a functional rip-stop. Then, also using a free needle, the FiberTape loop sutures are passed through the abductor tendons proximal to the FiberWire core sutures. Each of FiberTape loop suture will be incorporated into the final suspension bridge construct.

**Fig. 5:**
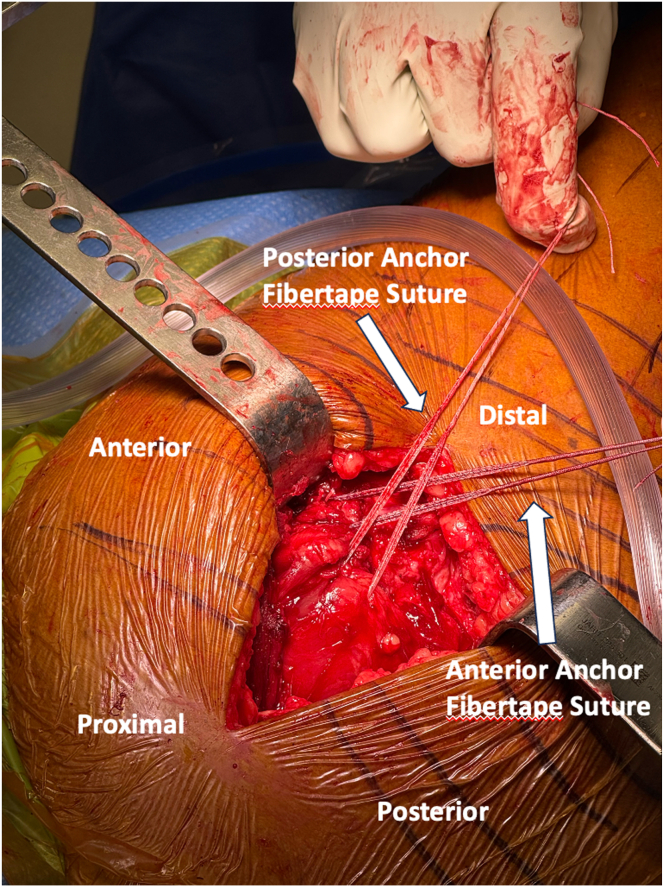
Superior view of the right, lateral hip with patient in the lateral decubitus position, deep self-retaining retractor retracting iliotibial band anteriorly and posteriorly. The hip is brought into slight abduction with the assistance of the Mayo stand, and the FiberWire core sutures are tied. Once restoration of anatomic tendon footprint of the gluteus medius and minimus tendons with adequate resting tension is confirmed, the distal row anchors may be established.

**Fig. 6:**
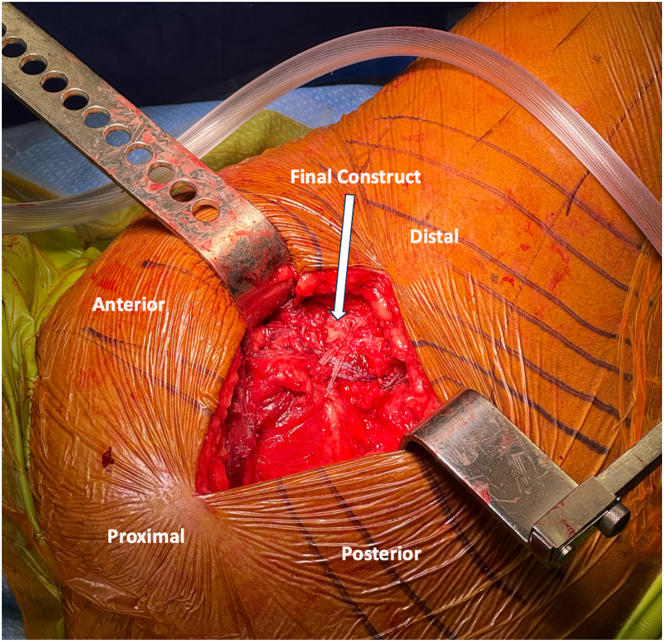
Superior view of the right, lateral hip with patient in lateral decubitus position, deep self-retaining retractor retracting iliotibial band anteriorly and posteriorly. The distal row anchors (2 × 4.75-mm vented PEEK SwiveLock anchors) are placed posteriorly and anteriorly along the vastus ridge in order to recreate the native anatomic footprint of the gluteus medius and minimus tendons, maximizing facet coverage. The corresponding FiberTape loop sutures from the gluteus minimus tendon are incorporated into the posterior, distal row anchor, and the FiberTape loop suture from the gluteus medius is incorporated into the anterior, distal row anchor. The repair is dynamically assessed with hip internal and external rotation, showing compression of the footprint and movement of a single repair unit.

**Fig. 7:**
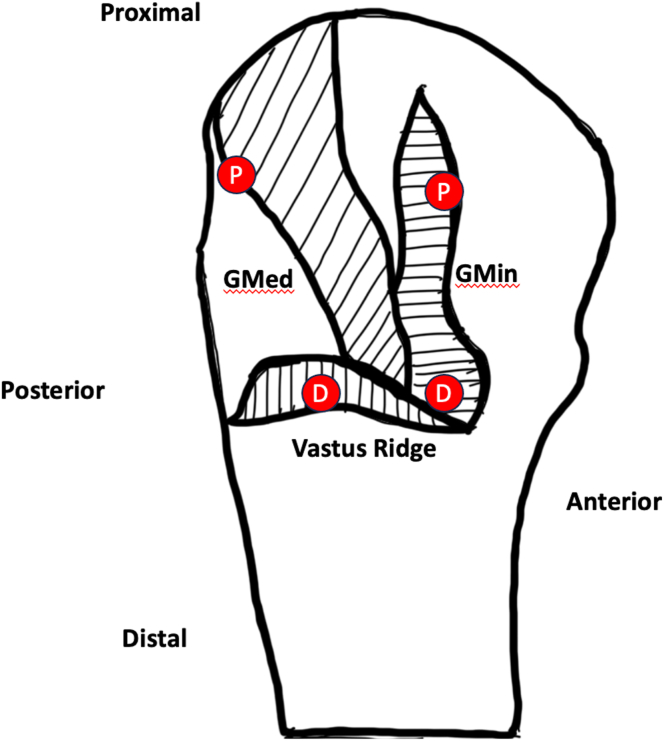
Anatomic rendering of the tendinous insertions onto the greater trochanter when viewed from a lateral perspective. Outlines of insertional footprints (gluteus medius, gluteus minimus, and vastus ridge) onto lateral aspect of greater trochanter are shown. Anatomic depiction of proximal row anchor placement, labeled “P”, and distal row anchor placement, labeled “D”, maximizing restoration of native anatomic footprint and facet coverage.

At this point, the double-row repair with rip-stop augmentation is begun. Proximally, 2 vented anchors (4.75-mm SwiveLock; Arthrex, Naples, FL) are placed into the anterior and posterior aspect of the greater trochanter after the bone is first prepared with a drill and tap (Fig 4, Video 1). The anchors are preloaded with a FiberTape loop and FibeWire core suture (Video 1). With a free needle, the FiberWire Core sutures are passed separately into respective gluteus medius and gluteus minimus tendons from superficial to deep (Video 1). The goal is to recreate an interior to posterior spread in horizontal mattress fashion to later serve as a functional rip-stop. Then, also using a free needle, the FiberTape loop sutures are passed through the abductor tendons proximal to FiberWire core sutures. Each of FiberTape loop suture will be incorporated into the final suspension bridge construct. At this point, the hip is brought into slight abduction with the assistance of the Mayo stand, and the FiberWire core sutures are tied (Fig 5, Video 1). Once restoration of anatomic tendon foot print with adequate resting tension is confirmed, the distal row anchors may be established (Video 1). The distal row anchors are placed posteriorly and anteriorly along the vastus ridge in order to recreate the native anatomic footprint of the tendons, maximizing facet coverage (Table 1, Video 1). The corresponding FiberTape loop sutures from the gluteus minimus tendon are incorporated into the posterior, distal row anchor, and the FiberTape loop suture from the gluteus medius is incorporated into the anterior, distal row anchor (Figs 6 and 7, Video 1). The repair is dynamically assessed with hip internal and external rotation, showing compression of the footprint and movement of a single repair unit (Video 1). Following copious irrigation with saline, the limb is placed into internal rotation and neutral adduction for ITB closure with braided, absorbable suture (Video 1). Deep and subcuticular dermal layer is then closed with absorbable suture (Monocryl; Ethicon, Somerville, NJ) and topical skin adhesive (Dermabond; Ethicon). Sterile dressing is applied (Aquacel; ConvaTec, Princeton, NJ), and the patient is extubated with subsequent transfer to postanesthesia care unit in stable condition.

Postoperatively, the patient is restricted to partial weight bearing to 20 lbs with a walker and placed into a hip abduction brace (Ossur, Foothill Ranch, CA) for 6 weeks (Video 1). Afterward, the patient may transition to full weight bearing by week 8 with progressive range of motion and isometric exercises starting 5 days after surgery with no resistive abduction exercises for at least 12 weeks. Physical therapy is begun at 6 weeks and typically continued for 6 to 8 months postoperatively.

## Discussion

Abductor tendon tears are increasingly recognized as a cause of recalcitrant lateral hip pain, historically attributed to trochanteric bursitis. Advances in imaging and histologic analysis, revealing a lack of bursitis in these patients, have led to more aggressive evaluation and operative treatment.^5^^,^^6^

Endoscopic repair, typically performed by hip arthroscopy specialists, allows concurrent treatment of intra-articular pathology with improved cosmesis.^18^^,^^21^^,^^26^ The procedure involves incising, debriding, and reapproximating tendons via suture anchors. Early data from high-volume centers report low failure rates and complications at mid-term follow-up.^24^^,^^26^^,^^27^ Despite endoscopic advantages, open repair remains favored in select cases based on surgeon expertise and tear morphology. Open techniques provide superior visualization of the anatomic footprint, precise tendon assessment, and enhanced fixation with larger suture bites, more passes, and improved bony preparation.^28^^,^^32^ Variations in open repair techniques include transosseous tunnels, suture anchors, synthetic or allograft augmentation, and muscle transfer reconstruction.^25^^,^^29^ However, most evidence consists of case series and retrospective studies, lacking randomized trials to guide optimal technique selection.^30^^,^^31^

Parallels exist between abductor and rotator cuff repair debates.^23^ Biomechanical studies demonstrate superior footprint coverage and higher load to failure with DR suture constructs compared to SR, although clinical outcomes remain inconclusive.^22^ This debate informed our use of the DR, rip-stop technique, previously described in rotator cuff repair for its advantages in suture pullout resistance, footprint restoration, and load sharing.^33^, ^34^, ^35^ While DR, rip-stop repair has yet to prove superior to SR in short-term clinical outcomes, improved structural healing rates have been reported.^36^ This technique offers a secure, reproducible method for anatomic abductor tendon repair.

This procedure has risks, including suture anchor complications, infection, retear, suboptimal pain, or functional improvement. Patients, particularly those with significant preoperative fatty atrophy and retraction, should be counseled on realistic expectations. Although the described technique is effective and reproducible, future prospective, randomized trials are needed to determine the optimal open abductor repair approach.

## Disclosures

The authors (J. J. R., C.C.A., H.B.G.B., E.J.H., W.M.P.) declare that they have no known competing financial interests or personal relationships that could have appeared to influence the work reported in this paper.

## Supplementary Data

Video 1 Open hip abductor repair using double row rip-stop technique. Below is a detailed step-by-step legend of the procedure described in the video, with time stamps referenced throughout. The perspective of each frame is that of the operative surgeon, standing at the posterior hip of the patient positioned in a lateral decubitus position with the patients distal, ipsilateral lower extremity to the right within the viewing frame. **0:00 – Introduction** •The technique for open hip abductor repair using a double row rip-stop method is introduced. The authors have no relevant disclosures. **0:08 – Incision and Exposure** •The operation is performed in the lateral decubitus position for optimal soft tissue retraction. A 10-cm longitudinal incision is made over the greater trochanter with a #10 blade. Dissection continues through skin and subcutaneous tissue to the deep fascia and iliotibial band (ITB). **0:31 – ITB and Bursa Exposure** •The ITB is incised longitudinally in line with its fibers and the femur’s axis, exposing the trochanteric bursa. **0:40 – ITB Tension Relieved by Padded Mayo Stand** •A padded Mayo stand is used for the operative foot, providing abduction and relieving ITB tension as needed. **0:50 – Retraction and Visualization** •Self-retaining deep retractors are placed. The extremity is internally rotated to visualize the anterolateral and posterosuperior footprints of the gluteus medius and minimus. **1:02 – Bursa Excision and Debridement** •The greater trochanteric bursa is excised, revealing the typical “bald” trochanter with large, retracted tears. Mucoid degenerated tissue is debrided to mobilize the torn abductor tendons. **1:16 – Tendon Mobilization** •An Alice clamp is used to mobilize the torn tendon edges, confirming excursion and resting tension. Traction stitches are placed to aid mobilization. **1:27 – Footprint Preparation** •The tendon footprints are prepared with Cobb elevators, curettes, and Rongeur to create a bleeding bone bed, facilitating tendon-to-bone healing. **1:40 – Double Row Rip-Stop Repair Begins** •Two vented anchors are placed proximally into the anterior and posterior greater trochanter after drilling and tapping. Anchors are preloaded with FiberTape loop and FiberWire core suture. **2:00 – Anchor Security and Suture Passage** •After anchor placement, security is verified by pulling the sutures. If there is an anchor pullout, a larger anchor is used. Using a free needle, the surgeon passes the FiberWire core sutures into the gluteus medius and minimus tendons in a horizontal mattress fashion, serving as a functional rip-stop. FiberTape loop sutures are passed to the abductor tendons proximal to the FiberWire core sutures. **2:34 – Construct Formation** •Each FiberTape loop suture will be incorporated into the final suspension bridge construct. The hip is slightly abducted with the Mayo stand, and the FiberWire core sutures are tied. Restoration of the anatomic footprint and tension is confirmed. **2:56 – Distal Row Anchor Placement** •Distal row anchors are placed posteriorly and anteriorly along the vastus ridge to recreate native tendon footprints and maximize facet coverage. FiberTape loop sutures from the gluteus minimus go into the posterior distal row anchor; sutures from the gluteus medius go into the anterior distal row anchor. **3:23 – Suture Loading and Trial Reduction** •Sutures are loaded into the distal row fixation anchor. Trial reduction is performed to the vastus ridge to ensure coverage and estimate anchor placement. The distal row is drilled and taped at the chosen location. **3:36 – Final Tensioning and Repair Assessment** •Sutures are tensioned to compress the footprint as they are incorporated into the distal row anchor. The repair is dynamically assessed with hip internal and external rotation, confirming compression and movement as a single repair unit. Tissue quality is checked to determine whether graft augmentation is needed. **4:02 – Closure** •After copious saline irrigation, the limb is placed into internal rotation and neutral adduction. The ITB is closed with braided absorbable suture. Deep and subcuticular dermal layers are closed with absorbable suture. A sterile dressing is applied. **4:19 – Postoperative Protocol** •The standard postoperative protocol is referenced. Patients remain partial weight bearing to 20 pounds with a walker. A hip abduction brace is worn for 6 weeks. Transition to full weight bearing at 8 weeks. Progressive range of motion and isometric exercises begin 5 days postoperation. No resisted abduction exercises were done for 12 weeks. Physical therapy is begun at week 6 and continued for 6-8 months postoperation. The video concludes with a summary and well wishes for using the technique to treat hip abductor tears.
